# Regulatory T Cells Control Immune Responses through Their Non-Redundant Tissue Specific Features

**DOI:** 10.3389/fimmu.2013.00294

**Published:** 2013-09-23

**Authors:** Sari Lehtimäki, Riitta Lahesmaa

**Affiliations:** ^1^Turku Centre for Biotechnology, University of Turku and Åbo Akademi University, Turku, Finland

**Keywords:** regulatory T cell, tissue specificity, systems biology, tolerance, immunity, microbiota

## Abstract

Regulatory T cells (Treg) are needed in the control of immune responses and to maintain immune homeostasis. Of this subtype of regulatory lymphocytes, the most potent are Foxp3 expressing CD4+ T cells, which can be roughly divided into two main groups; natural Treg cells (nTreg), developing in the thymus, and induced or adaptive Treg cells (iTreg), developing in the periphery from naïve, conventional T cells. Both nTreg cells and iTreg cells have their own, non-redundant roles in the immune system, with nTreg cells mainly maintaining tolerance toward self-structures, and iTreg developing in response to externally delivered antigens or commensal microbes. In addition, Treg cells acquire tissue specific features and are adapted to function in the tissue they reside. This review will focus on some specific features of Treg cells in different compartments of the body.

## Introduction

Regulatory T cells (Treg) maintain immune homeostasis, prevent autoimmune and allergic responses, and control the magnitude and duration of inflammatory responses. Although there are other cell types that also participate in these processes and are sometimes named Treg cells, such as IL-10 producing Tr1 cells and TGF-β producing Th3 cells, this review will use the term Treg cells only for the most potent regulators, i.e., CD4+ T cells expressing Forkhead box P3 transcription factor (Foxp3) (1).

Foxp3 has been shown to be the key regulator of Treg cell differentiation and function, as demonstrated by the devastating effects of loss-of-function mutations in the Foxp3 gene, leading to the lethal IPEX syndrome (immune dysregulation, polyendocrinopathy, enteropathy, X-linked). Patients with this disorder suffer from severe autoimmune responses, persistent eczema, and colitis. Similar effects can also be observed in mice with mutated Foxp3 (2–7).

Treg cells can be divided into two major subsets, natural Treg (nTreg) cells, which develop in the thymus, and induced Treg (iTreg) cells, which develop in the periphery from naïve T cells in response to such signals such as low immunogenic doses of antigen, commensal microbes, lymphopenia, or activation by immature DCs (8–11). In addition, iTreg cells can also be generated under inflammatory conditions, as has been shown in infections with certain pathogens, and mouse disease models, in which the development of the disease is due to the absence of nTreg cells (12).

To date, no unique marker that can distinguish nTreg cell from iTreg cell has been found. It has been suggested that the intracellular molecule Helios, which does not seem to be involved with the suppressive potential of Treg cells, is expressed only by nTreg cells in non-immunized animals (13). However, under certain activating conditions, Helios is also expressed by iTreg cells (14, 15), and in humans, a small subset of naïve Foxp3+ cells with suppressive function can lack Helios expression (16). Another potential marker that might distinguish these two cell types is Neuropilin-1 (Nrp-1), which also seems to be expressed only by nTreg cells (17, 18).

Treg cells often accumulate at the site of inflammation, most likely to control the magnitude and the duration of the inflammation, and in this way protect the host from immune-mediated pathology (19–23). Foxp3+ Treg cells have been reported to express transcription factors associated with effector T cells, including the Th1 transcription factor T-bet, Th2 transcription factor Gata3, and the Th2- and Th17-cell-related transcription factor IRF4 (24–26). These probably facilitate the expression of a number of features that are shared with effector T cells, such as the same homing receptors, which would allow Foxp3+ Treg cells to localize to the same site with the cells they are expected to suppress.

All of the environmentally exposed areas of the human body, such as the gastrointestinal tract, respiratory tract, skin, and urogenital tract, are covered by commensal microbiota (27). In addition to this, these surfaces are continuously facing harmless antigens, such as pollen or food. One of the main functions of Treg cells is the induction of tolerance toward these innocuous agents. Failure to do so may lead to development of allergy or inflammatory responses against commensals, which can be detrimental to the host (7, 10, 28). However, pathogens also utilize mucosal sites and the skin to enter the body. In order to be able to mount an appropriate immune response for a given trigger, the immune system constantly needs to balance between tolerance and immunity.

Tolerogenic dendritic cells (tDC) and structural cells, such as pulmonary stromal cells or intestinal epithelial cells (IEC), promote tolerance by producing anti-inflammatory substances, such as TGF-β, retinoic acid (RA), and TSLP (29), which are known to promote Treg generation and function. In addition, at least in the gut and in the lungs, a specific subset of macrophages seems to induce Treg cells (30–32).

Treg cells are also found in those compartments of the body that are not directly exposed to external environment, where they most likely mainly participate in the maintenance of immune homeostasis and prevention of autoimmunity. It is not surprising that these Treg cells possess several features that distinguish them from Treg cells that are continuously exposed to external antigens. A recent report by Feuerer et al. (33) nicely demonstrated the transcriptional similarities and differences between Treg cells from lymph nodes (LN), spleen, adipose tissue, and Treg cells induced under different conditions. These comparisons clearly show that although they are all suppressive in nature and clearly closer to each other than to conventional T cells, they all have unique features of their own. These observations combined with mouse models, where it is possible to follow effects of either nTreg cell or iTreg cell depletion or deficiencies in homing receptors of Treg cells in different disease models, indicate that multiple Treg cell subsets have their own specific roles in the immune system and although they have overlapping tasks, they are also needed to complement each other’s functions (19, 24, 34–36). In this review we will go through some of the specific features of Treg cells in defined compartments of the body (Table 1) and how Treg cells in these different sites contribute to tolerance.

**Table 1:** **Features of Treg cells in different compartments of the body**.

Site	Special features	Main function for Treg cells
Gut	High number of iTreg cells induced by orally delivered antigens and commensal microbes	Oral tolerance (systemic)
Skin	High number of Treg cells in the steady state. UV-radiation induced Treg cells	Immune homeostasis
Lung	Pathogens affect Treg number and function	Induction of tolerance against nasal antigens
Liver	Antigen presentation in the liver may lead to formation of iTreg cells which confer systemic tolerance	Systemic tolerance
Fat tissue	Limited TCR repertoire	Control of sterile, low-grade inflammation in the adipose tissue

## Treg Cells in the Gut

Over 60 years ago, Chase (37) reported that the feeding of an antigen resulted in an “immunologic non-responsiveness” to the same antigen when it was later administered systemically (37). This kind of non-responsiveness to intestinal antigens is now referred to as oral tolerance (38, 39). In mice, oral tolerance can be transferred from one animal to another through adoptive transfer of CD4+CD25+ cells (40, 41). On the other hand, depletion of Foxp3+ cells abolishes the oral tolerance (31), indicating that tolerance is not solely a result of effector T cell depletion or anergy but is also actively induced through activation of Treg cells.

In addition to orally administered antigens, tolerance to gut microbiota has been reported several years ago by Powrie et al. (42) who demonstrated that adoptive transfer of CD4+CD45RB^high^ cells into immunodeficient mice resulted in the development of colitis, and the inflammatory response could be prevented by simultaneous transfer of CD4+CD45RB^low^ cells, i.e., Treg cells. Colitis was not provoked in germ-free (GF) mice indicating that inflammatory response against commensal microbes was involved in the pathology (43, 44). Treg cells do develop in GF-mice and are able to suppress T cell proliferation, but according to some experiments, not as efficiently as Treg cells from conventionally housed mice (45, 46). Recent studies indicate that colon derived microbes play an important role in shaping the T cell repertoire in the gut and certain bacterial species seem to be particularly efficient in promoting Treg cell differentiation and suppressive function (8, 47). At least some of the gut derived Treg cells have TCRs recognizing gut microbe derived antigens. Moreover, adoptively transferred colon microbe recognizing Treg cells with a GFP marker, localized preferentially in the colon (48). Interestingly, iTreg cells also shape the microbial colonies in the gut. In mice devoid of iTreg cells, the gut microbiota is different from WT mice, possibly due to spontaneous Th2 type inflammation developing in the gut in the absence of iTreg cells (24).

Several studies indicate that a large proportion of gut Treg cells are derived from conventional T cells, which are converted into immunosuppressive Foxp3+ cells and are thus iTreg cells. Adoptive transfer of Foxp3^−^ OT-II T cells into mice subsequently fed with OVA resulted in expansion of OT-II specific Foxp3^+^ cells (31). Additionally, in mice where nTreg development was inhibited but iTreg cells could form, administration of OVA in the drinking water lead to formation of OVA-specific iTreg cells and prevented subsequent sensitization with OVA (19, 40). When the development of both of these cells was prevented, no tolerance was generated (19). A great majority of gut derived Treg cells lack Helios and Nrp-1 expression, indicating that they are not thymic derived Treg cells (13, 17, 48). Finally, the TCR repertoire of Treg cells in the gut has been shown to be different from Treg cells in the secondary lymphoid organs, indicating development in the periphery from naïve precursors (48). This observation has, however, been challenged in a recent paper by Cebula et al. (49), who found a significant overlap between TCRs of gut derived and thymus derived Treg cells. Over 90% of the TCRs of gut derived Treg cells, which were able to recognize gut commensal antigens, were also expressed by Foxp3+ thymocytes. Based on these observations, plus the fact that colitis is not induced in CNS1^−/−^ mice devoid of iTreg cells (24), and that the colonization of GF-mice with a specific microbiota results in the expansion of nTreg cells (50), the authors concluded that nTreg cells constitute the majority of gut Treg cells and dominantly confer tolerance to gut microbiota. It is true that adoptive transfer of Foxp3− cells into lymphopenic host induces colitis in the recipient, although a certain proportion of the transferred cells convert into Foxp3 expressing iTreg cells (10). Moreover, *in vitro* generated and adoptively transferred iTreg cells were unable to cure the colitis in this same model, although they did prevent the lethality of the disease. However, nTreg cells were unable to cure the colitis either, unless transferred together with iTreg cells or transferred into mice that could generate iTreg cells. These results indicate that nTreg cells and iTreg cells have non-redundant roles in tolerance against gut microbiota. Furthermore, CNS1^−/−^ mice develop spontaneous Th2 type inflammation in the gut (24), and conversion of Foxp3− into Foxp3+ cells has been observed upon colonization of GF-mice with specific microbiota (50). As a conclusion, most likely iTreg cells, but also nTreg cells participate in the tolerance against gut microbiota. High interpersonal variability in the gut’s microbial communities has been observed, whereas at the individual level, the variation is minimal over time (51, 52), and therefore tolerogenic responses against gut microbes could resemble tolerance toward self-structures. Instead, iTreg could be especially important for generating tolerance to orally delivered antigens with even more diverse and “fluctuating” antigen repertoire. This view is supported by OVA feeding experiments, as described above as well as the study by Nagatani et al. (53) indicating that adoptively transferred OVA-specific naïve T cells in Peyer’s patches (PP) start to express Foxp3 after oral administration of ovalbumin.

Induced Treg cells in the gut are most likely induced in the mesenteric LNs (mLN) by tolerance inducing CD103+ DCs. Synthesis of RA and the production of immunosuppressive indoleamine-2,3-dioxygenase (IDO) by these DCs are involved in the induction (54–56). Intestinal macrophages, which arise from different precursors than DCs, may also play a role in the Foxp3 conversion and/or proliferation in the gut (30–32). In addition, IEC participate in the induction of oral tolerance by secreting TSLP, TGF-β, and RA, and thus creating a local microenvironment which favors tolerogenic responses, for instance by enabling DCs to produce RA from food derived vitamin A (29). Microbes may also favor conversion, for example, bacteria derived matrix-metalloproteinases may cleave latent TGF-β into active form which supports Treg conversion. Moreover, *Bacteroides fragilis* and *Clostridium* species, both gut microbiota, have been shown to promote the number and function of certain Treg cell subsets in the gut (8, 47).

Establishment of oral tolerance requires migration of tDCs into to mLN to activate Foxp3 cells. Mice devoid of mLN are unable to mount oral tolerance (57) and an impaired inhibition of colitis has been observed in mice deficient of CCR7 or CD62L (58, 59), most likely due to impaired homing of Treg cells to the mLNs. However, subsequent recruitment of Foxp3 cells into the lamina propria (LP) and local expansion there is also necessary for the establishment of oral tolerance. Foxp3+ cells proliferate only little in the mLN but vigorously in the LP. In addition, oral tolerance is weaker or abolished in mice with deficiencies in gut homing molecules, such as integrin α4β7+ or chemokine receptor CCR9+ expressed on Treg cells or mucosal addressin cell adhesion molecule-1 (MAdCAM1) expressed on intestinal venules and PPs (31, 60). It has been speculated that this local expansion may offer an additional checkpoint between immunity and tolerance (31).

## Treg Cells in the Skin

Skin is continuously exposed to a variety of external antigens, pathogens, and chemicals. The structure of the skin forms a physical barrier to prevent harmful substances or antigens entering the body. In addition, many chemical and biochemical properties, such as low pH, low water content, production of antimicrobial peptides, or composition of lipid compounds, protect the host from the “outside invaders.” However, if a pathogen/chemical is able to breach the epithelial barrier and penetrate into the skin, both the innate and the adaptive immune systems are activated (61, 62).

Like the gut, also skin is covered by commensal bacteria, which in humans occupy specific niches, like sebaceous glands or hair follicles (63). Furthermore, as in the gut, the skin commensals are needed for the development of a proper immune response in the skin, and commensals have been shown to modulate especially IL-1 driven inflammation (64). Although gut induced tolerance has been shown to have systemic effects (39), absence or reduction of gut commensal bacteria does not affect skin commensals or their ability to modulate immune responses in the skin (64).

In the steady state, both the skin Langerhans cells (LCs) and dermal dendritic cells (dDCs) maintain the tolerance through induction/activation of Treg cells. While LCs have been shown to promote skin resident Treg cell proliferation (65), it seems that mainly dDCs present self-antigens in the skin draining LNs (66–68). Approximately 6% of DCs in the skin draining LNs in the mouse have been shown to originate from the skin in the steady state (23). Vitamin D plays a role in the development of tolerogenic DCs in the skin. Vitamin D3 is activated by UV-radiation and vitamin D receptor agonist treatment of DCs elicits Treg inducing tDCs and LCs (69–71). In addition, RA producing DCs, which in contrast to gut are CD103−, have been shown to induce Tregs in the skin draining LNs (72), although their number is lower in the dermal LNs compared with intestinal tract (73). During primary response to an antigen, iTreg cells are generated from conventional T cells in the skin draining LNs (74).

In the steady state, skin has a high proportion of Treg cells and they are needed to maintain local homeostasis in the skin. This becomes evident in experiments where Treg recruitment into the skin is impaired, e.g., due to the absence of skin homing chemokine receptor, CCR4, or E/P-selectin ligand, α-1,3-fucosyltransferase VII (23, 75–77). Although Treg cells deficient in these molecules are functional in *in vitro* suppression assays and are able to control other peripheral autoimmune responses in *scurfy* mice, they are not able to control spontaneous inflammation developing in the skin. However, during hapten induced contact hypersensitivity (CHS) response, Treg cells accumulate in the skin even in the absence of CCR4 receptor (78), possibly through another chemokine receptor such as CCR10 (78, 79) or CCR5, which is important for Treg recruitment into the skin during *Leishmania major* infection (80). CCR7 and CCR6 expression have also been reported in skin derived Treg cells. Other markers typical for skin resident Treg cells are CD44 and CD103, indicating that the majority of skin Treg cells are of memory/effector type (23, 75, 76).

During CHS response, Treg cells migrate from the skin exposed to hapten to the draining LN. Upon rechallenge with the same hapten, they migrate back to the skin, into the site of new exposure (23). These skin derived Treg cells are more suppressive than LN derived Treg cells and they function, at least partly, in an antigen-specific manner. In addition, they express more IL-10, TGF-beta, and have higher surface expression of CD103 and GITR (23). Increasing percentages of Treg cells accumulate in the skin at different time points after challenge and ablation of Treg cells during the challenge phase of CHS results in a prolonged inflammation, pointing to an important role for Treg cells in terminating the inflammatory response at the site of inflammation (21, 23).

UV-irradiation of the skin induces Treg cells, which can prevent sensitization to contact allergen in an antigen-specific manner when adoptively transferred from UV radiated and hapten exposed mouse to a naïve recipient prior to sensitization (81). These UV-iTreg cells express Foxp3, CD4, CD25, CTLA-4, GITR, neuropilin, and CD62L (82). The mechanism for Treg induction involves LCs, which suffer from UV radiation in such a way that they are unable to present hapten professionally, resulting in tolerance instead of immunity. Keratinocytes may also be involved through RANK-RANKL mediated mechanisms (83, 84). UV-iTreg cells are, however, unable to suppress the elicitation phase in an already sensitized recipient, unless injected directly at the site of hapten administration. This is most likely due to expression of LN homing molecule, CD62L, on Treg cells, which prevents their migration into the skin. Education of these UV-iTreg cells *in vitro* or *in vivo* with skin derived APC equips the cells with skin homing molecules, ligands for E-and P-selectin, and results in the suppression of the elicitation phase. These results clearly demonstrate that UV-iTreg cells can efficiently suppress both primary and secondary responses and that they are needed at the site of inflammation, i.e., the skin, during the secondary response (82). Like Treg cells in general, UV-iTreg cells are also a heterogeneous population, possessing different properties depending on the UV-dosage, mouse strain, and even hapten used to sensitize the mice (85).

## Treg Cells in the Lung

In the lungs, normal flora and exogenous antigens delivered via the airways trigger DCs and lung resident tissue macrophages to induce iTreg cells in order to control pulmonary homeostasis and tolerance (29, 86, 87). Moreover, pulmonary stromal cells participate in the process by promoting differentiation of tDCs (88).

Low doses of antigen administered intranasally induce generation of Foxp3+TGF-β+ cells, which maintain tolerance to the same antigen when later administered at doses that would induce sensitization (89). Foxp3+ cells control allergic airway inflammatory responses and their depletion during the sensitization phase results in increased IgE titers and eosinophilia after challenge in the lungs (20, 90). Adoptive transfer of Treg cells in the challenge phase, in turn, results in suppressed inflammatory response, and both nTreg cells and *in vitro* generated iTreg cells are capable of suppression (91–93). Importantly, mice with defects in iTreg generation develop spontaneous inflammation in the lungs (and gut) with features of Th2 type allergic airway inflammation (24).

Mice with CCR4 deficient Treg cells develop spontaneous lung inflammation (76) and in contrast to the skin, efficient Treg recruitment into the lungs during inflammation also requires CCR4 (35). Moreover, asthmatic patients show increased percentages of CCR4+ Foxp3+ Treg cells in the BAL fluid after allergen challenge. CCR4 expression seems to be especially important during secondary allergic inflammatory responses, whereas in the priming phase, Treg mediated suppression requires CCR7 expression (34) and allergic airway inflammation is exacerbated in the absence of CCR7 (94).

Pathogens affect Treg cells in the lungs and may have consequences on tolerance. While some infections increase the number of Treg cells, promote their function, and subsequently inhibit sensitization to common allergens, some infections exacerbate allergic inflammation in the lung. For instance, vaccination of mice with bovis Bacille Calmette–Guérin (BCG) or recombinant BCG expressing house dust mite protein Derp, reduced subsequent allergic airway inflammation after OVA or Derp sensitization and challenge, respectively. In addition, increased numbers of Foxp3+ cells in the lung and enhanced expression of CTLA-4 and elevated levels of IL-10 and TGF-b were observed (95, 96). Instead, infection early in life with respiratory syncytial virus (RSV) impaired Treg function and exacerbated allergic airway inflammation in mice (97).

## Treg Cells in the “Inside”

Tissues not directly exposed to outside world, such as organs and adipose tissue also harbor Treg cells with specific functions. A couple of examples of these sites are considered, as follows.

The liver favors immune tolerance and it has been already known for a long time that liver allografts are accepted even without immune suppression or require less immunosuppression for long term survival than other transplants (98, 99). Furthermore, liver-allograft recipients seem to tolerate non-hepatic allografts from the same donor, and ongoing rejection can be reversed by liver allografts, indicating a systemic tolerant response (100). Although the liver is not directly exposed to environmental antigens, it is continuously sampling food and microbial antigens from the gut and may also play a role in the development of oral tolerance (101). In addition, being a primary metabolic organ, several neo-antigens are developed during metabolic processes, which may require tolerance. Several cell types in the liver, such as liver sinusoidal endothelial cells, Kupffer cells, stellate cells, and DCs, can present antigens in the context of suppressive cytokines and inhibitory cell surface molecules and promote tolerance (102). Although several mechanisms of tolerance prevail, such as clonal deletion or immune deviation (103–105), increasing evidence also points toward the role of Treg cells in mediating hepatic tolerance. For instance, in a mouse model of Concavalin A (ConA) induced T cell mediated liver injury, Treg cell depletion results in more severe inflammation, whereas adoptive transfer of Treg cells ameliorates the symptoms. These hepatic Treg cells express more Foxp3, CTLA-4, CD103, and GITR than their splenic counterparts, and are more suppressive *in vivo* than Treg cells from non-treated mice (106, 107). More importantly, targeted expression of antigens in the liver can establish systemic tolerance by inducing antigen-specific Foxp3+ Treg cells (108, 109). For example, in a mouse model of multiple sclerosis, ectopic expression of neuronal autoantigen myelin basic protein (MBP) in the liver prevented the development of the disease. The protection could be transmitted to a WT recipient through adoptive transfer of Foxp3+ Treg cells from mouse expressing MBP in the liver. Treg cells were shown to develop from conventional T cells in a TGF-b dependent manner and only when the autoantigen was expressed in the liver, not in the skin (109).

Obesity is accompanied by low-grade inflammation in the adipose tissue, which has consequences on insulin-resistance and subsequently, development of type 2 diabetes (110, 111). In normal mice, Foxp3+ cells can be found in the adipose tissue, but in the obese insulin-resistance mouse models or mouse fed with high fat diet, the number of these cells is reduced (112, 113). Loss-of-function/depletion experiments and gain-of-function/adoptive transfer experiments, demonstrate that these Foxp3+ cells are responsible for controlling the inflammation in the fat tissue and development of insulin resistance (113, 114). In mice, T cells comprise approximately 10% of the abdominal fat tissue cells, of which three quarters are CD4+ T cells, and approximately half of these express Foxp3, which is a high percentage compared to lymphoid and non-lymphoid tissues. Interestingly, high Fopx3+ cell numbers are recovered from visceral adipose tissue, but not from subcutaneous tissue, the former being associated with insulin resistance (110, 113). The TCRs of fat deposited Treg cells are distinct from their LN resident counterparts and have a limited TCR repertoire, indicating that fat Treg cells may be selected through antigen specificity. Also the transcriptional signature of adipose derived Treg cells is different from Treg cells derived from secondary lymphoid organs or Treg cells induced *in vivo* or *in vitro* (33). In humans, both lowered Foxp3 expression (112, 113) and increased Foxp3 expression in the adipose tissue (115) has been shown to accompany obesity. Eller et al. (114), in turn, observed similar Foxp3 mRNA expression in obese patients with insulin resistance, whilst Foxp3 expression in obese patients without insulin resistance was lowered, compared to healthy controls. The expression of Helios was decreased in both obese groups, indicating lowered nTreg numbers. Clearly further studies are needed to confirm Treg contribution to fat tissue inflammation in humans.

## Genome-Wide Analysis

Feuerer et al. (33), have demonstrated that the gene-expression in different murine Treg cells differs from that of conventional T cells. However, when comparing, for example, spleen derived Treg cell signatures with fat tissue derived Treg cells or with Treg cells induced *in vivo* or *in vitro*, it becomes evident that Treg cells form a heterogeneous group of suppressive cells. The rapid development of technologies enabling fast and reproducible genome-wide analysis of the cells has enabled finding such small differences between the subsets of cells. These methods include array- or next-generation sequencing based transcriptome studies, proteomic studies and ChIP-Seq analysis, which enable detection of novel protein coding and non-coding transcripts, differential splicing, epigenetic modifications, or transcription factor-binding sites along the whole genome [reviewed in Ref. (116)]. Genome-wide approaches provide valuable information about complex networks occurring in the cells during or in response to differentiation and reveal novel genes and mechanisms participating in these processes. For example, array based analysis of differentiated human Th1 and Th2 cells revealed several previously unknown genes differently expressed between these cell types (117, 118). Similarly, analysis on human cord blood derived Th1/Th2/Th17 cells during the early differentiation process identified several new candidates potentially involved in the fate decision of these cells (119–121). Functional analysis of these candidates in turn, resulted in the discovery of new regulators of human Th cell differentiation (122–126). Genome-wide approaches have also been utilized to unravel the role of transcriptional regulators, such as STAT6, SATB1, or ATF3, in the differentiation process (122, 124, 127). Similarly in mouse, genes regulated by STAT4 and STAT6 during Th1 or Th2 cell development have been studied (128, 129), and quite surprisingly, only a small subset of genes was similarly regulated by STAT6 in mouse and human, underlying the importance of human studies.

A large proportion of the single-nucleotide polymorphisms (SNPs) associated with autoimmune diseases identified through GWAS studies are in the non-coding regions of DNA. To investigate how such SNPs may participate in molecular mechanisms of human diseases, integrative analysis of human cells is required. For example, a recent study that combined analysis of transcription factor-binding sites, chromatin state maps, modeling of enhancer-gene pairs, and genome-wide association studies of autoimmunity-associated SNPs provide a potential link for distal regulatory regions in disease pathogenesis. Such SNPs were shown to alter binding of transcription factors involved in Th cell differentiation (130).

Several systems biology approaches have also been used to study Treg cells, including genome-wide analysis of Foxp3 binding sites, both in mouse and human (131–134), transcriptomes of resting and activated nTreg and iTreg cells in mice (33, 135, 136) and epigenetic modifications of Treg cells in mouse and human (137–139).

Whilst the regulatory functions of Treg cells indicate their potential therapeutic use, there are a large number of questions that should be answered first. For instance, the stability of Treg cells is one of the major concerns. Natural Treg cells have been shown to prevent onset of different autoimmune diseases in several animal models and have already been tested also in clinical trials (140, 141). Additionally, a number of studies have demonstrated a remarkably more demethylated Foxp3 locus in nTreg cells compared to iTreg cells, which is thought to provide a more stable suppressive phenotype in nTreg cells (137, 142, 143). However, in some studies, iTreg cells have shown enhanced stability over nTreg cells during inflammation, due to IL-2 and TGF-β -mediated downregulation of IL-6 receptor, which makes iTreg cells more resistant to conversion into Th17 cells. In addition, in contrast to nTreg cells, iTreg cells also retained suppressive activity *in vivo* in inflammatory conditions (144–146). These results suggest that while nTreg cells might be efficient in preventing the onset of the disease, iTreg cells could be more suitable for treating the already established disease.

The heterogeneity of Treg cells and their non-redundant roles in immune tolerance also imply that the mechanisms of suppression may differ between different Treg types, tissues, and inflammatory conditions. Also antigen specificity plays a role. The expansion of nTreg cells *in vivo* or *in vitro* generates a polyclonal pool of cells, whereas iTreg cells can be generated with a certain antigen specificity, which may diminish harmful side effects. Finally, while having a lot of information of more or less terminately differentiated Treg cells, we still lack information about gene-expression and regulation during Treg development, especially in humans. Genome-wide studies, including transcriptomes, regulomes, and proteomes, will help us to recognize the subtle differences between different Treg subtypes, identify the factors that drive the differentiation process toward Treg phenotypes and define the elements that contribute to the stability and functionality of these cells. In the future this knowledge could be used to effectively manipulate and generate functional Treg cells for therapeutic purposes.

## Conclusion

Treg cells have attracted intense attention, due to their therapeutic potential for a number of different conditions including autoimmune diseases, allergies, transplantation, and even obesity related diseases, such as type 2 diabetes. In this review we were able only to scratch the surface of different qualities of Treg cells in different tissues, but even this short glimpse makes it easy to realize that Treg cells have multiple functions in the body, with unique requirements at any given site. It is therefore understandable that the Treg cells that govern all these processes comprise a heterogeneous group of cells with distinct tissue specific features.

To study such small differences in a mixed group of cells, holistic approaches are needed. Several systems biology studies concerning different effector Th cell subsets, including Treg cells, have been conducted. So far, the majority of these genome-wide approaches regarding Treg cells have been animal studies, and it remains to be seen how many of these observations are translated to humans. We also lack information of gene-expression and regulation during early Treg differentiation. The more we know about different Treg subsets and the factors that promote their development, the more targeted therapies can be designed, including homing of Treg cells exactly where they are needed and differentiating Treg cells in a way that assures optimal function in the target tissue. This will minimize the number of cells needed for therapies and increase the specificity, resulting in diminished harmful side effects like suppression of antitumor immunity or induction of chronic infections.

## Conflict of Interest Statement

The authors declare that the research was conducted in the absence of any commercial or financial relationships that could be construed as a potential conflict of interest.
